# Individuals Maintain Similar Rates of Protein Synthesis over Time on the Same Plane of Nutrition under Controlled Environmental Conditions

**DOI:** 10.1371/journal.pone.0152239

**Published:** 2016-03-28

**Authors:** Ian D. McCarthy, Stewart F. Owen, Peter W. Watt, Dominic F. Houlihan

**Affiliations:** 1 School of Ocean Sciences, Bangor University, Menai Bridge, Anglesey, United Kingdom; 2 Instituto Oceanográfico, Universidade de São Paulo, São Paulo, SP, Brazil; 3 AstraZeneca, Alderley Park, Macclesfield, Cheshire, United Kingdom; 4 Sport and Exercise Science and Medicine, University of Brighton, Eastbourne, United Kingdom; 5 School of Biological Sciences, Aberdeen University, Aberdeen, United Kingdom; Fred Hutchinson Cancer Research Center, UNITED STATES

## Abstract

Consistent individual differences in animal performance drive individual fitness under variable environmental conditions and provide the framework through which natural selection can operate. Underlying this concept is the assumption that individuals will display consistent levels of performance in fitness-related traits and interest has focused on individual variation and broad sense repeatability in a range of behavioural and physiological traits. Despite playing a central role in maintenance and growth, and with considerable inter-individual variation documented, broad sense repeatability in rates of protein synthesis has not been assessed. In this study we show for the first time that juvenile flounder *Platichthys flesus* reared under controlled environmental conditions on the same plane of nutrition for 46 days maintain consistent whole-animal absolute rates of protein synthesis (A_s_). By feeding meals containing ^15^N-labelled protein and using a stochastic end-point model, two non-terminal measures of protein synthesis were made 32 days apart (d_14_ and d_46_). A_s_ values (mass-corrected to a standard mass of 12 g) showed 2- to 3-fold variation between individuals on d_14_ and d_46_ but individuals showed similar A_s_ values on both days with a broad sense repeatability estimate of 0.684 indicating significant consistency in physiological performance under controlled experimental conditions. The use of non-terminal methodologies in studies of animal ecophysiology to make repeat measures of physiological performance enables known individuals to be tracked across changing conditions. Adopting this approach, repeat measures of protein synthesis under controlled conditions will allow individual ontogenetic changes in protein metabolism to be assessed to better understand the ageing process and to determine individual physiological adaptive capacity, and associated energetic costs of adaptation, to global environmental change.

## Introduction

Consistent individual differences (CID) [1] in physiology and behaviour drive individual fitness (*i*.*e*. survival and reproductive output) under variable environmental conditions and hence provide the framework through which natural selection can operate [2, 3, 4]. Underlying this concept is the assumption that in order for selection to be able to operate, individuals will display consistent levels of performance in fitness-related traits, i.e. they will maintain the same levels of performance and/or the same relative performance ranking over time compared to other individuals.

Although greater attention has focused on individual variation and broad sense repeatability (defined in [5] as ‘the extent to which individual differences in scores are maintained over time (in a given context)’) in behavioural performance such as personality/boldness, courtship/mate choice and parental behaviour (reviewed in [6]), some physiological traits have also been examined and there is growing interest in describing CIDs in physiological performance and understanding how individual physiological and behavioural performance traits interact to determine specific life-histories within the fast-slow pace of life continuum [1, 7]. Most studies that have examined CIDs in physiology have focused on locomotor performance (reviewed in [8]) and metabolic rate (reviewed in [9, 10]) given the obvious fitness-related effects of individual variation in these parameters with regards to predator evasion and food capture and in maximisation of energy allocation for growth and reproduction respectively. However, individual performance consistency has been assessed for a number of other physiological parameters such as metabolic enzyme activities [11], evaporative water loss [12–15], thermal conductance [15] and blood chemistry parameters [16, 17, 18], although these parameters have been less well-studied.

Proteins play a central role in maintenance (*i*.*e*. tissue replacement) and growth in animals through the processes of protein synthesis and breakdown [19] with previous studies highlighting how inter-individual differences in rates of protein synthesis and breakdown affect growth performance in molluscs [20, 21, 22] and fishes [23, 24, 25]. This work has shown that faster growing and/or more efficient individuals do so through having lower rates of protein turnover (equivalent to protein breakdown in growing animals) [26]. However, most studies of protein synthesis in non-mammalian animals have made single terminal measures using radiolabelled (^3^H- or ^14^C-labelled, but usually ^3^H-phenylalanine) amino acids using the flooding dose technique of Garlick et al. [27] (see reviews by Houlihan et al. [28] and Fraser and Rodgers [29] for application in fishes). Although the wide application of the flooding dose technique using ^3^H-phenylalanine across a range of non-human taxa enables direct comparison of data between studies within and across taxa [26, 28, 29], the drawback of this technique is that it provides a single terminal measure of protein synthesis precluding any studies of broad sense repeatability and so conclusions drawn on inter-individual differences in physiological performance are based on a single measure. In contrast, the use of stable isotopes, *i*.*e*. ^13^C- or ^15^N-labelled amino acids, is required in human studies, and is more widely used in mammalian studies of protein synthesis [30, 31]. In studies of human protein metabolism, repeat measures of protein synthesis using stable isotopes are common and, for example, longitudinal studies have tracked rates of protein synthesis for known individuals under different exercise regimens [32, 33] or dietary regimens [34], or before and after surgery [35] or the application of medical treatments [36]. However, despite the use of repeated measures of protein synthesis using stable isotopes in mammalian studies (*op*. *cit*.) and in some ectotherm studies (*e*.*g*. *Mytilus edulis* [37]; *Platichthys flesus*, [38]), broad sense repeatability, *per se*, has not been quantified.

Alternative stable isotope techniques for measuring rates of protein synthesis in fish have been developed using ^15^N-labelled protein [39, 40], deuterium oxide (^2^H_2_O) [41] or ^2^H-labelled amino acids [42, 43]. The advantage of these stable isotope methodologies is that they are either non-terminal as they determine protein synthesis through the measurement of whole-animal nitrogen-flux rates [39, 40] or offer the potential for non-terminal biopsy sampling [41, 42, 43] and therefore offer the possibility that repeat measures of protein synthesis can be made on known individuals. However, repeat measures in fish have only been used once, by Carter et al. [38], to examine seasonal changes in protein synthesis and growth in juvenile (18–60 g) flounder *Platichthys flesus* over 212 days. Although Carter et al. [38] made three repeat measurements on known individuals, the protein flux data at different times of the year were reported as group mean values to examine seasonal changes in protein metabolism and growth and quantifying individual variation and consistency was not the focus of the study. Thus, although inter-individual differences in protein metabolism in fishes are well documented [23, 24, 25], performance consistency and broad sense repeatability have not been assessed. However, since inter-individual differences in protein synthesis in fish can be due to differences in body size, plane of nutrition and abiotic factors such as temperature [28, 29, 44], these factors will need to be controlled for when seeking to determine individual consistency for this labile physiological trait. Therefore, the aim of this study was to assess individual consistency in rates of protein synthesis (mass-corrected to a standard size) using ^15^N-labelled protein in individual juvenile flounder *Platichthys flesus* reared under constant environmental conditions and maintained on the same plane of nutrition for 46 days.

## Materials and Methods

### Fish Husbandry

Eighteen juvenile flounder (*Platichthys flesus* L.) were caught using a hand-towed 1 m beam trawl from the intertidal stretches of the Tarty Burn within the River Ythan estuary (Aberdeenshire, UK; 57.3358°N, 2.0115°W) [45]. Each beam trawl tow lasted approximately 60 seconds and was towed for a distance of 30 to 50 metres in a water depth of approximately 30 cm in the Tarty Burn at low tide. At the end of each tow, juvenile flounder were quickly transferred to a bucket containing 25 litres of seawater aerated by a battery-powered air pump and all other animals in the net were quickly released back to the Tarty Burn. After 5 tows, 18 flounder of a suitable size were collected. Flounder were transferred to the seawater aquarium at Aberdeen University School of Biological Sciences (a journey time of approximately 30 minutes) where they were anaesthetized (MS222, 0.2 g l^-1^) [38] to determine initial mass (day 1, d_1_) and held individually in 10 L tanks (14°C, 33 psu; 12 h light: 12h dark photoperiod). The flounder (mean±SD initial mass 10.02±1.91 g, range = 6.34–12.91 g) were fed daily a single meal of ragworm *Nereis virens* (supplied by Seabait Ltd., Northumberland, UK) approximating to 4% of their wet body mass each day to maintain the fish on the same plane of nutrition during the experiment: this daily ration was offered to the fish at the same time of day (between 9 a.m. and 10 a.m.) and was always consumed by each fish on each day. Rates of protein synthesis were determined using a non-terminal ^15^N technique (after Carter et al. [38]; Carter et al. [39]; with additional modifications described below) on days 14 (d_14_) and 46 (d_46_). No mortality or disease symptoms were observed during the course of the study.

### [^15^N] methodology

Two non-terminal measures of protein synthesis were made 32 days apart (d_14_ and d_46_) using the stochastic end-point model [38, 39, 46]. Uniformly labeled ^15^N-algal protein (0.041g 90% Atom Percent Excess; Martek BioSciences Corporation, MD, USA) was first mixed thoroughly with porcine gelatine (0.107 g; Sigma) and then mixed with 500 μL distilled water. Pellets (2 mm diameter x 5.0 mm long) were made by setting the gelatine in silicon tube and cut to length under a dissection microscope. Each labeled gelatine pellet was inserted into the washed gut of a section of *Nereis* of known mass to provide a labeled meal (*ca*. 4% body mass d^-1^) for each flounder. Reference gelatine and *Nereis* were prepared without the addition of ^15^N in the same manner. Five unlabeled and five labeled preparations were selected at random, frozen (-20°C), freeze dried (-60°C; 48 hours), ground and each preparation sampled in triplicate by combustion mass spectrometry (see below). The *Nereis* meals were labelled at 0.879±0.008 (n = 5) Atom Percent Excess. The mean coefficient of variation (CV) of the five triplicates of the enriched food delta was 0.5% (range 0.4–2.0%) and the CV of the unlabelled food delta was 4% (range 0.7–10.8%). The protein content of the labelled meals was 7.29±0.03% (n = 15) of the wet mass as determined from the total N content of the sample from mass spectrometry data using a conversion factor of 5.85 as the ratio of N:protein [47]. Protein consumption on d_14_ and d_46_ were expressed on an absolute basis (A_r_) as mg protein d^-1^.

On d_14_ and d_46_, the same experimental protocol was followed (except for the timings of water sampling on d_46_, see below). Labeled *Nereis* meals were fed to individual flounder (between 9 a.m. and 10 a.m.); care was taken to ensure that the food was consumed whole. Individual flounder were then transferred to 1.5 L of aerated water where they remained for 72 h (d_14_) or 48 h (d_46_) (see below) with regular water changes. During water changes (see below for timings), 30 mL samples were taken in order to measure the excretion rate of ammonia-Nitrogen (A_N_) using an ion-selective electrode (Unicam, UK). At the same sampling times, 1 L samples were collected and acidified (5 ml of 2M HCl) for isotope analysis. The ammonia was trapped in boric acid [26, 39], frozen (-20°C), freeze dried (-60°C, 7 d) and duplicate borate samples analysed for ^15^N-ammonia enrichment by combustion in a Carlo Erba NA 1500NC sample converter linked to a Micromass Optima Isotope Ratio Mass Spectrometer. The rate of whole-body protein synthesis was calculated from the rate of protein flux through the body less the total nitrogen excretion as described in Carter et al. [38] and Carter et al. [39]. On d_14_, excretion and isotope abundance samples were collected at 6, 12, 24, 36, 48, 60 and 72 h following the meal. On d_46_, a second determination of whole-body protein synthesis was made using the same methodology as on d_14_ except isotopic samples were only collected and total ammonia-N excretion was only measured collected 24 and 48 h after the meal (see results). Whole-body rates of protein synthesis were calculated on an absolute basis (A_s_) as mg protein synthesised d^-1^.

### Ethics statement

No specific permits were required to collect the animals and the sampling procedures were not subject to review or approval prior to collection. Animals were collected using a standard field sampling technique. The species collected in this study is not endangered or protected. The work was conducted in 1995 before the introduction of University Ethical Review Committees at UK universities and therefore was not subject to approval or review by a University Committee. The experimental work was conducted under licence from the UK government Home Office under Animals (Scientific Procedures) Act 1986.

### Statistical analysis

All data in this study can be found in S1 File. Statistical comparisons and curve fitting were performed using SPSS v20.0 (SPSS Inc, USA). Data are presented as mean values ± one standard deviation (SD). The cumulative rate of isotope excretion (e*) was expressed as a percentage of ^15^N in the meal (dose) and described using the equation e* = a(1-e^-kt^) where t is time after feeding and a and k are constants [39]. The rate of ammonia excretion (A_N_, μg N g^-1^ h^-1^) was described using the equation A_N_ = ae^-bt^ where t is time after feeding and a and b are constants [48]. Differences in the cumulative rate of isotope excretion and rate of ammonia-N excretion over time were examined using repeated-measures ANOVA to identify the period of constant isotope excretion rate and constant ammonia excretion, and hence allow flux and synthesis calculations to be made with least overestimate [39, 46]. Since individual fish increased significantly in body mass during the 46 day experiment (see results), absolute rates of protein synthesis on d_14_ and d_46_ were scaled to a standard 12 g fish A_s(std)_ using the equation A_s(std)_ = A_s(obs)_*(12/M_obs_)^0.74^ [20] where A_s(obs)_ and M_obs_ are the absolute rate of protein synthesis (mg d^-1^) and body mass (g) for an individual fish and 0.74 is the mass-exponent for protein synthesis [49]. Absolute rates of protein consumption on d_14_ and d_46_ were similarly mass-corrected for a 12 g flounder using a mass-exponent of 0.75 [50, 51]. Anabolic stimulation of protein synthesis on d_14_ and d_46_ was calculated from the mass-corrected data as A_s_/A_r_ (mg protein synthesized mg^-1^ protein consumed). Mass-corrected absolute rates of protein synthesis and protein consumption and A_s_/A_r_ values for each fish on d_14_ and d_46_ were compared using paired *t* tests. In addition, individual temporal consistency of rates of protein synthesis on the same plane of nutrition was assessed using interclass correlation. Although broad sense repeatability is traditionally assessed using the intraclass correlation coefficient [52, 53], in studies where only two measures of performance are being compared the interclass correlation coefficient (Pearson’s product-moment correlation, *r*) is used as an estimate of broad sense repeatability over time [54, 55, 56]. Interclass correlation assesses the consistency of a trait relative to the mean, *i*.*e*. examines whether individuals are consistently placed within the population distribution [55].

## Results

**Fig 1:**
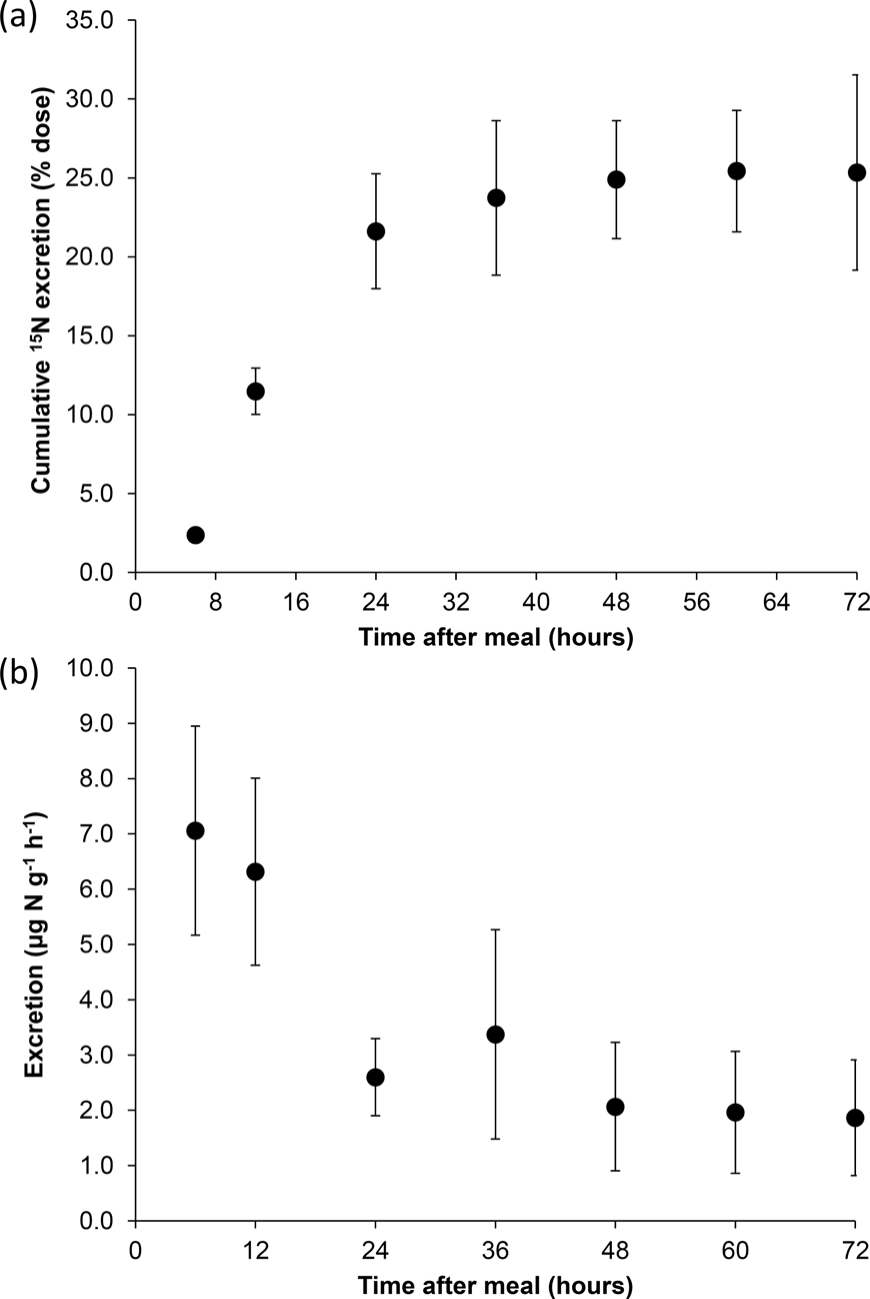
Validation of stochastic end-point model to measure rates of protein synthesis in *P*. *flesus* on d_14_. (a) The mean (± SD) cumulative excretion of ^15^N (expressed as a percentage of the dose) and (b) the mean (± SD) ammonia-nitrogen excretion (μg N g^-1^ body mass h^-1^) over 72 hours after feeding a 4% body mass meal of *Nereis virens* labeled with ^15^N to juvenile flounder *Platichthys flesus* (11.74 ± 1.98 g, n = 18).

On d_14_, cumulative ^15^N excretion (Fig 1A) was described by e* = 27.36(1-*e*^-0.049t^) (*r*^*2*^ = 0.93, n = 7, *P* < 0.001) with a constant rate of isotope excretion attained 24h after feeding the labelled meal. Comparison of the cumulative rate of isotope excretion revealed significant differences 6–72 h post-feeding (repeated-measures ANOVA, *F*_6,125_ = 118.1, *P* < 0.001) however, rates were similar between 24–48 h post-feeding (Tukey HSD test, all *P* > 0.05). Therefore, the rate of isotope appearance was constant 24–48 h post-feeding indicating a steady state had been achieved and on d_46_ isotopic samples were only collected at 24 and 48h after feeding. On d_14_, ammonia excretion rates were determined for each individual and the total ammonia excretion calculated for the 72 h period following feeding the labeled meal (Fig 1B). Ammonia excretion rates were described by A_N_ = 8.01e^-0.027t^ (*r*^*2*^ = 0.87, n = 7, P < 0.001). Comparison of ammonia excretion rates 6–72 h post feeding revealed significant differences (repeated-measures ANOVA, *F*_6,125_ = 76.4, *P* < 0.001) however, rates were similar between 24–48 h post-feeding (Tukey HSD test, all *P* > 0.05). Therefore, constant rates of ammonia excretion and cumulative isotopic excretion 24–48 h after feeding the labeled meal allowed the stochastic end-point model to be used to calculate individual rates of protein synthesis between 24 and 48 h after feeding the labeled meal.

**Fig 2:**
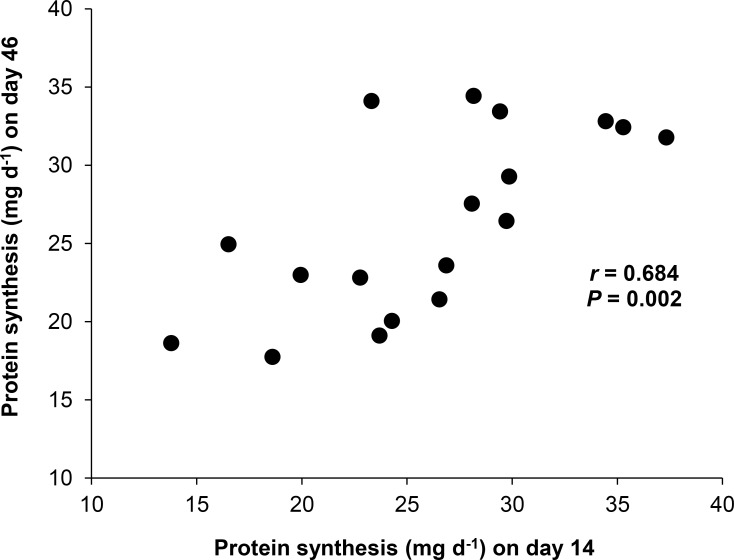
Consistency in rates of protein synthesis in *P*. *flesus* maintained on the same plane of nutrition for 46 days. Mass-corrected absolute rates of protein synthesis (A_s_, mg protein synthesized d^-1^) of juvenile flounder *Platichthys flesus* (n = 18) on days 14 (11.74 ± 1.98 g) and 46 (13.63 ± 2.43 g). A_s_ values have been corrected to a standard mass of 12 g. Broad sense repeatability represented by the Pearson’s product-moment correlation coefficient is indicated on the plot.

The 18 juvenile flounder increased in size over the 46 day experiment and individual body masses on d_14_ and d_46_ were significantly different from each other (Table 1). As a result the protein consumption and synthesis data were mass-corrected to a standard mass of 12 g. Although individual flounder were feeding at the same plane of nutrition (*ca*. 4% body mass d^-1^ ration) over the 46 day experiment, whole animal growth rates varied between 14.5 and 91.9 mg wet mass d^-1^ (mean growth rate 44.9 ± 21.4 mg wet mass d^-1^). For both protein consumption and protein synthesis, mass-corrected absolute rates on d_14_ and d_46_ were similar (*P* > 0.05; Table 1). Therefore, the anabolic stimulation of protein synthesis on d_14_ and d_46_ were not significantly different (Table 1; *P >* 0.05) with on average 1.13 mg protein synthesized per mg protein consumed. A significant positive correlation was found between the mass-corrected absolute rates of protein synthesis on the two measurement days (Fig 2; *r* = 0.684, *P* = 0.002) indicating significant consistency in individual rates of protein synthesis measured on d_14_ and d_46_ for fish maintained on the same plane of nutrition for 46 days under controlled environmental conditions.

**Table 1:** Absolute rates (mg d^-1^) of protein consumption (A_r_) and protein synthesis (A_s_) and anabolic stimulation (A_s_/A_r_, mg protein synthesised per mg protein consumed) for juvenile flounder *Platichthys flesus* (n = 18). A_r_ and A_s_ data have been corrected to a standard mass of 12 g.

	d_14_	d_46_	Paired *t* test (17 *df*)
**Mass (g)**	11.74 (± 1.98)	13.63 (± 2.43)	*t* = 8.92
	[8.54–15.36]	[9.63–18.92]	*P* < 0.001
**A**_**r**_ **(mg d**^**-1**^**)**	23.5 (± 5.5)	23.9 (± 5.0)	*t* = 0.31
	[13.3–30.9]	[16.5–33.0]	*P* = 0.76
**A**_**s**_ **(mg d**^**-1**^**)**	26.0 (6.4)	26.3 (5.8)	*t* = 0.25
	[13.8–37.3]	[17.8–34.4]	*P* = 0.81
**A**_**s**_**/A**_r_*	1.12 (0.18)	1.13 (0.31)	*t* = 0.16
	[0.82–1.48]	[0.77–1.92]	*P* = 0.87

* = calculated from mass-corrected values

## Discussion

This study has utilized the stochastic end-point method of Waterlow et al. [46] (as modified by Carter et al. [39]) to measure *in vivo* whole-animal rates of protein synthesis. Our validation data show that between 24 and 48 hours post-feeding both ammonia excretion and cumulative isotope excretion rates were constant, as reported in other studies using the same methodology [38, 39, 57, 58, 59]. Fish appear well suited to the application of the stochastic end-point model as labelled nitrogen appears in a single major excretory product, ammonia, which on average accounts for approximately 80–90% of total N excreted with the remaining excreted primarily in the form of urea [60, 61]. Although urea was not measured in the present study, previous studies on a range of teleost species that have used this method to measure rates of protein synthesis have concurrently measured rates of ammonia (A_N_) and urea (U_N_) excretion and isotope labelling of these excretory products. These studies [38, 39, 57, 58, 59] have shown that rates of urea excretion are low (U_N_ equal to 15.7 ± 9.1% of total nitrogen excretion (T_N_) assuming that T_N_ = A_N_ + U_N_) and fall within the values expected for teleost fishes [60, 61]. In addition, studies using the same methodology have shown that urea remains unlabelled up to 72 h after feeding the uniformly-labelled protein [57, 59] indicating that the nitrogenous waste derived from the labelled food is excreted solely in the form of A_N_. Ammonia-N is the primary end-product for nitrogenous excretion derived from amino acid oxidation whereas urea-N is primarily derived from pyrimidine and purine base excretion [59, 62].

**Fig 3:**
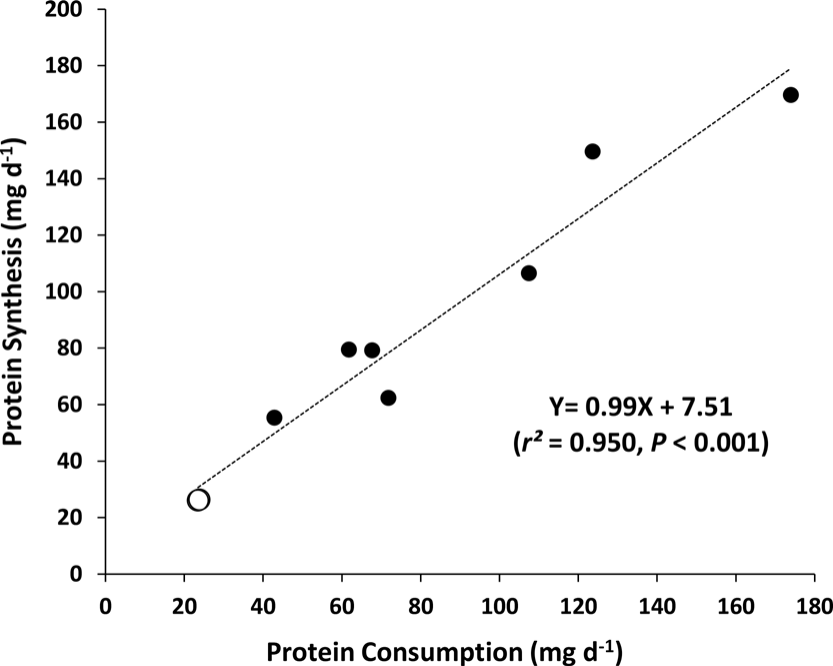
Effect of protein consumption on protein synthesis in fishes at 14–16°C. The relation between mass-corrected whole-animal absolute rates of protein consumption (A_r_, mg protein d^-1^) and protein synthesis (A_s_, mg protein synthesized d^-1^) for various teleost fish species at 14–16°C. Original consumption and synthesis data have been mass-corrected to a standard mass of 12 g. Information on species, actual body mass and source reference are presented in S1 Table. Data from the present study are indicated by the large open circles on the plot.

The absolute rates of protein synthesis for juvenile flounder measured in this study are at the magnitude expected at this temperature given the plane of nutrition of the fish (Fig 3; Data presented in S1 Table), providing confidence in the methodology and the resulting protein synthesis data. Fig 3 summarises the available data on absolute rates of protein consumption and protein synthesis (both mass-corrected to a 12 g fish) in fishes reared between 14 and 16°C. As expected, both diet quality and quantity affect the rate of protein synthesis with most studies feeding formulated feeds with a high protein content and having higher rates of protein synthesis compared to the present study where natural food (ragworm) with a lower protein content was fed to juvenile *P*. *flesus* (S1 Table). Given the lower rate of protein consumption, rates of protein synthesis measured in the present study are low but are at the magnitude expected given the plane of nutrition of the fish and the relation between absolute rates of protein consumption and protein synthesis for fish at 14–16°C (Fig 3). Since Houlihan et al. [63] reported that the relation between absolute rates of protein consumption and protein synthesis in Atlantic cod *Gadus morhua* was 1:1, the anabolic stimulation of protein synthesis (synthesis/consumption; [28]) in fishes has been the focus of some research interest and a range of anabolic stimulation values have been reported ranging from 0.89 to 1.29 milligrammes of protein synthesized per milligramme of protein consumed (see S1 Table, plus data presented in Carter and Houlihan [64]) with the juvenile flounder in the present study presenting anabolic stimulation values of 1.12 and 1.13 mg mg^-1^ respectively (Table 1). This range of anabolic stimulation values observed may be related to differences in diet composition between studies, as the level of anabolic stimulation (and subsequent retention efficiency) in salmonids has been shown to be related to the protein:energy ratio in the diet [64]. Overall, the relation between A_r_ and A_s_ for fish at 14–16°C (Fig 3) indicates that on average 0.99 milligrammes of protein are synthesized per milligramme of protein consumed, the same level of anabolic stimulation as proposed by Houlihan et al. [63]. This level of anabolic stimulation is lower compared to endotherms where the data presented by Houlihan et al. [26] for 6 endotherm species suggests that stimulation ratios are much higher (1.58 mg protein synthesized mg^-1^ protein consumed, range 1.01–2.72 mg mg^-1^; data from Fig 2 in Houlihan et al. [26]) with a large proportion (77%, range 64.4–84.4%) of synthesized protein used for protein turnover, i.e. to replace existing body protein). This high level of protein turnover, and subsequent recycling of amino acids into the free amino acid pool in endotherms may account for the higher anabolic stimulation ratio per unit protein consumption.

In the present study, individual variation in mass-corrected whole-animal rates of protein synthesis showed a 2.7-fold variation between the lowest and highest rates observed on d_14_ and a 1.9-fold variation on d_46_ respectively (Table 1). This observed individual variability in rates of protein synthesis in fishes is not uncommon with previous studies reporting 2.1 to 5.0 fold variation in whole-animal synthesis [23–25, 63]. However, it is interesting to note that although the variability observed in previous studies may, in part, be due to differences in body mass or consumption rate (but see McCarthy et al. [25] for individual variability in fish of a similar size on the same plane of nutrition), the present study shows that even controlling for body mass and plane of nutrition, individuals can still show considerable variation in rates of protein synthesis. This has been attributed to genotype-dependent differences in maintenance requirements that drive individual differences in protein turnover and growth efficiency [65, 66].

The results of this study show that individual juvenile flounder reared under controlled environmental conditions and maintained on the same plane of nutrition maintain similar mass-corrected absolute rates of protein synthesis with an estimated broad sense repeatability of 0.68. Repeat measures are routine in studies of human protein metabolism (e.g. [32, 33, 34, 36]) but there are only two published studies in ectotherms [37, 38]. Where repeat measures have been made, data are usually presented as measures of central tendency with an associated measure of dispersion (usually mean ± standard deviation) and analysed using a repeated-measures ANOVA to compare differences in group average responses across treatments or over time having controlled for repeat measures made on the same individuals [32, 34, 38]. However, there are two studies, on *M*. *edulis* [37] and on humans [67], where individual protein synthesis data are presented (Table 1 in [37]; Table 3 in [67]) allowing broad sense repeatability to be calculated (see S2 Table). Hawkins et al. [37] present repeat measures of absolute rates of protein synthesis (mass-corrected for animals of 1 g dry tissue weight) made on twelve *M*. *edulis* acclimated to 10°C followed by an acute temperature transfer to 20°C with a second measurement made 48 hours later. Analysing their data no performance consistency in rates of protein synthesis can be observed (*P* > 0.05, S2 Table), however, the data may not be directly comparable as a result of the effects of abrupt temperature transfer on mussel physiology, individual differences in thermal sensitivity and the observed differences in feeding rates at 10 and 20°C [37]. Heys et al. [67] measured fractional rates of protein synthesis in replicate biopsy samples taken from malignant breast tumours in nine human subjects (see S2 Table). These concurrent measures of protein synthesis were significantly repeatable (*P* < 0.001, S2 Table) with an intra-class correlation coefficient [53] of 0.88 (with 1 indicating perfect repeatability; S2 Table). Thus, the available data, although limited, indicate significant repeatability in rates of protein synthesis measured concurrently [67] and significant temporal repeatability in animals maintained on the same plane of nutrition (the present study).

**Fig 4:**
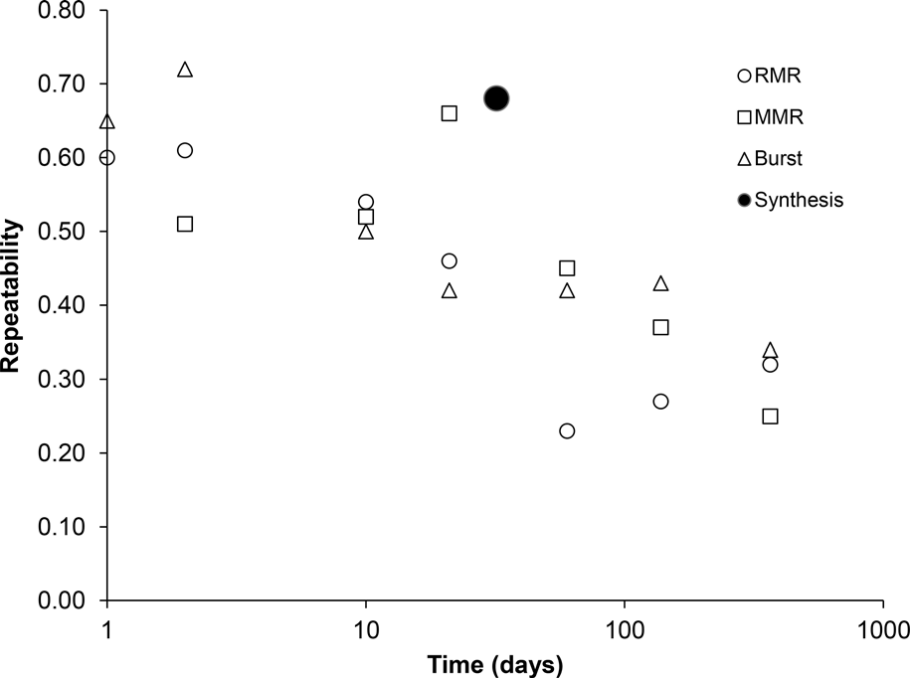
Temporal changes in repeatability of physiological performance. The effect of increasing time interval between repeat measures on the broad sense repeatability of routine metabolic rate (RMR), maximum metabolic rate (MMR) and burst locomotor performance (Burst) plus an estimate of broad sense repeatability for absolute rates of protein synthesis over 32 days calculated from the results of the present study. Metabolic rate data are derived from S1 Table of White et al. [10] and locomotor performance data are derived from S2 Table of Laming et al. [8] respectively and are presented as average values for the following time intervals: < 1 day, 1–2 days, 1–2 weeks, 2–4 weeks, 1–3 months, 3 months– 1 year and > 1 year. These data are plotted on the abscissa as values of 1, 2, 10, 21, 60, 138 and 365 respectively. Raw data used in the plot can be found in S1 File.

Most studies that assess consistency of physiological performance have focused on metabolic rate and locomotor performance although some other physiological measures such as blood chemistry parameters, evaporative water loss and metabolic enzyme activities have also been assessed for CIDs and performance consistency (see Introduction for references). Broad sense performance repeatability is reported to decline over time for metabolic rate (reviewed by White et al. [10]) and for burst locomotor performance (reviewed by Laming et al. [8]). Fig 4 provides a summary of the decline in average broad sense repeatability over time for routine and maximum metabolic rate and for burst locomotor performance in ectotherms and endotherms: average values for defined time intervals (see Figure legend for details) were calculated from data collated in S1 Table of [10] and S2 Table of [8]. Short-term (i.e. < 2 weeks) repeatabilities for these physiological parameters average values of *ca*. 0.5 to 0.7 (although individual studies range from -0.1 to 0.98) with medium term (*i*.*e*. months) and long term (*i*.*e*. ≥ 1 year) repeatability declining to average values of *ca*. 0.3 to 0.5 (range = -0.10 to 0.90) and 0.3 to 0.4 (range = 0.02 to 0.47) respectively (see [8, 10] for data) (Fig 4). The repeatability value calculated from the present study for absolute rates of protein synthesis in juvenile flounder falls within the range of broad sense repeatability values reported for physiological performance over the time scale of 28 to 42 days (0.12 to 0.79; see [8, 10] for data), although it is at the higher end of the data range. Biro and Stamps [5] have recently highlighted how ignoring time-related changes on both between- and within-individual performance in labile behavioural and physiological traits can result in either invalid or biased (*i*.*e*. underestimated) estimates of broad sense repeatability. These time-related changes may include the effects of changing environmental conditions (e.g. temperature, photoperiod), time of day, feeding history, size, age, sex and maturity [5]. Biro and Stamps [5] comment that estimates of broad sense repeatability tend to be low with meta-analyses of labile behavioural and physiological traits providing mean estimates of 0.4 as result of these temporal effects. Interestingly, the average values for the datasets presented in Fig 4 are 0.43, 0.46 and 0.50 for routine metabolic rate, maximum metabolic rate and burst locomotor performance respectively. The higher level of broad sense repeatability observed in the present study may be a reflection of controlling for potential confounding factors such as environmental conditions (temperature, photoperiod, salinity), size, plane of nutrition and time of day (when fed and when the experimental measurements were taken) in assessing broad sense repeatability. However, repeatability in the present study did not approach unity (i.e. r = 1) indicating that not all time-related lability may have been accounted for, e.g. effects of sex, age, differential temporal changes between individuals in endogenous rhythms of physiological performance or responses to the stress of handling/confinement during measurement. Alternatively, as recently highlighted by Biro and Stamps [5], it is likely that the small sample size (n = 18) and low level of replication (n = 2) in the present study are insufficient to determine the global broad sense repeatability for this physiological trait, although the results do indicate a high level of consistency within this group of experimental animals, and future studies will need to increase both of these factors. In addition, it is interesting to note that performance consistency is not always maintained across different environmental conditions [68] or following significant life-stage transitions such as metamorphosis [69] which may help explain why CIDs and phenotypic variation persist within species as this will always allow some individuals to perform well within a fluctuating environment.

In conclusion, this study has used a non-terminal end-point method using ^15^N-labelled protein [39] to determine the consistency of mass-standardized rates of protein synthesis in juvenile flounder and has shown, for the first time in fish, that individuals maintained under controlled environmental conditions (temperature, photoperiod, salinity) on the same plane of nutrition for 46 days maintain similar rates of protein synthesis with a broad sense repeatability estimate of 0.68. To the authors’ knowledge, this is the first attempt to estimate broad sense repeatability for this fundamental metabolic process. The use of the non-terminal methodologies in studies of animal ecophysiology should be encouraged where possible as this will allow the individual to be the unit of replication in studies rather than relying on discrete groups of experimental animals. For example, the use of repeat measures of protein synthesis in fishes will allow the performance of known individuals to be tracked across changing experimental conditions (e.g. temperature, salinity, diet quality and quantity), to assess ontogenetic changes in protein metabolism in order to better understand the ageing process, and to better understand the adaptive capacity and energetic costs of adaptation to global climate change and ocean acidification. However, as highlighted recently by Biro and Stamps [5] care should be applied that sample sizes and levels of replication are sufficient to determine robust measures of performance consistency.

## Supporting Information

S1 File McCarthy et al Flounder data.xlsx. Excel data file containing the raw data used in this study.
(XLSX)

S1 Table Protein metabolism data for fish at 14–16°C. Mass-corrected whole–animal absolute rates of protein consumption (A_r_, mg protein d^-1^) and protein synthesis (A_s_, mg protein d^-1^) for juveniles of various fish species at 14–16°C. Data presented or calculated from the original source have been mass-corrected to a standard mass of 12 g according to Hawkins et al. [37].
(DOCX)

S2 Table Repeatability data for protein synthesis. A summary of published studies [37, 67] where repeat measures of protein synthesis have been made and where data are presented for individuals allowing repeatability to be calculated.
(DOCX)
