# Supplementary material for: Individuals Maintain Similar Rates of Protein Synthesis over Time on the Same Plane of Nutrition under Controlled Environmental Conditions
Source: PLoS One. 2016 Mar 28;11(3):e0152239. doi: 10.1371/journal.pone.0152239 (PMC4809500; doi:10.1371/journal.pone.0152239)
Supplement: S1 Table — Mass-corrected whole–animal absolute rates of protein consumption (Ar, mg protein d-1) and protein synthesis (As, mg protein d-1) for juveniles of various fish species at 14–16°C. Data presented or calculated from the original source have been mass-corrected to a standard mass of 12 g according to Hawkins et al. [37]. (DOCX) [file pone.0152239.s002.docx]

**Table S1. Protein metabolism data for fish at 14-16°C.** Mass-corrected whole–animal absolute rates of protein consumption (A_r_, mg protein d^-1^) and protein synthesis (A_s_, mg protein d^-1^) for juveniles of various fish species at 14-16⁰C. Data presented or calculated from the original source have been mass-corrected to a standard mass of 12 g according to Hawkins et al. [37].

| **Species** | **Mass (g)** | **T°C** | **Ration** | **A_r_*** | **A_s_*** | **Reference** |
| --- | --- | --- | --- | --- | --- | --- |
| **Flounder^1^** | 11.6 | 14 | 4% BM d^-1^, Natural food, 7.3% Protein | 23.5 | 26.0 | Present study^1^ |
| ***Platichthys flesus*** | 13.7 | 14 | 4% BM d^-1^, Natural food, 7.3% Protein | 23.9 | 26.3 | Present study^1^ |
|  | 26.7 | 14.9 | 2% BM d^-1^, Formulated food, 40.9% Protein | 42.9 | 55.4 | Carter et al. [38] ^1^ |
| **Greenback Flounder^1^** | 39 | 16 | 1% BM d^-1^, Formulated food, 48.3% Protein | 67.7 | 69.2 | Carter and Bransden [58] ^1^ |
| ***Rhombosolea tapirina*** | 37 | 16 | 2% BM d^-1^, Formulated food, 48.3% Protein | 123.7 | 149.6 | Carter and Bransden [58]^1^ |
| **Rainbow trout^2^** | 100 | 14 | 1% BM d^-1^, Formulated food, 43.3% Protein | 61.8 | 79.5 | Carter et al. [39] ^1^ |
| ***Oncorhynchus mykiss*** | 100 | 14 | 1% BM d^-1^, Formulated food, 43.3% Protein | 62.4 | 71.8 | Carter et al. [39] ^1^ |
|  | 305 | 14 | 2.7% BM d^-1^, Formulated food, 47.0% Protein | 174.0 | 169.7 | Dobley et al. [70]^1^ |
| **Atlantic salmon^2^** | 283 | 14 | 2% BM d^-1^, Formulated food, 47.4% Protein | 107.5 | 106.5 | Carter et al. [24] ^2^ |
| ***Salmo salar*** |  |  |  |  |  |  |

BM = Body Mass

* – corrected to a standard mass of 12 g.

1 – protein synthesis measured ^15^N-labelled protein (see Carter et al. [39])

2 – protein synthesis measured using ^3^H-phenylalanine (see Houlihan et al. [28])

70. Dobley A, Martin, SAM, Blaney SC, Houlihan DF**.** Protein growth rate in rainbow trout (*Oncorhynchus mykiss*) is negatively correlated to liver 20S

proteasome activity. Comp Biochem Physiol A. 2003, 137: 75-85
