# Supplementary material for: Individuals Maintain Similar Rates of Protein Synthesis over Time on the Same Plane of Nutrition under Controlled Environmental Conditions
Source: PLoS One. 2016 Mar 28;11(3):e0152239. doi: 10.1371/journal.pone.0152239 (PMC4809500; doi:10.1371/journal.pone.0152239)
Supplement: S2 Table — A summary of published studies [37, 67] where repeat measures of protein synthesis have been made and where data are presented for individuals allowing repeatability to be calculated. (DOCX) [file pone.0152239.s003.docx]

**Table S2. Repeatability data for protein synthesis**. A summary of published studies where repeat measures of protein synthesis have been made and where data are presented for individuals allowing repeatability to be calculated

| **Species** | **Tracer** | **Sample** | **No. of repeat** | **Study design** | **Repeatability** | **Reference** |
| --- | --- | --- | --- | --- | --- | --- |
|  |  | **size** | **measures** |  | **calculation** |  |
| Blue mussel | ^15^N-labelled | 15 | 2 | Acute temperature transfer experiment. | *r*_13_= 0.245^1^ | Hawkins et al. [37] |
| *Mytilus edulis* | Protein |  |  | Acclimated to 10**°**C for 21 days before | *P* > 0.05 | (Data presented in |
|  |  |  |  | first measure followed by abrupt |  | their Table 1) |
|  |  |  |  | transfer to 10**°**C with second measure |  |  |
|  |  |  |  | made 48h after transfer. |  |  |
| Man | ^13^C-Leucine | 9 | 2 (n= 6) | Variability in rates of protein | r = 0.88^2^ | Heys et al. [67] |
| *Homo sapiens* |  |  | 3 (n= 3) | synthesis measured in 2 or 3 | *P* < 0.001 | (Data presented in |
|  |  |  |  | concurrently sampled biopsies |  | their Table 3) |
|  |  |  |  | from breast tumours |  |  |

1 – repeatability reported as interclass correlation coefficient (Pearson’s *r*) as only two repeat measures of performance.

2 - repeatability reported as intraclass correlation coefficient where n_0_ is calculated as 2.32 [53].
